# Priming Strategies for Benefiting Plant Performance under Toxic Trace Metal Exposure

**DOI:** 10.3390/plants10040623

**Published:** 2021-03-25

**Authors:** Alina Wiszniewska

**Affiliations:** Department of Botany, Physiology and Plant Protection, Faculty of Biotechnology and Horticulture, University of Agriculture in Kraków, Al. 29 Listopada 54, 31-425 Kraków, Poland; a.wiszniewska@urk.edu.pl

**Keywords:** chemical priming, induced acclimation, priming cocktail, soil pollution, toxic metals and metalloids

## Abstract

Combating environmental stress related to the presence of toxic elements is one of the most important challenges in plant production. The majority of plant species suffer from developmental abnormalities caused by an exposure to toxic concentrations of metals and metalloids, mainly Al, As, Cd, Cu, Hg, Ni, Pb, and Zn. However, defense mechanisms are activated with diverse intensity and efficiency. Enhancement of defense potential can be achieved though exogenously applied treatments, resulting in a higher capability of surviving and developing under stress and become, at least temporarily, tolerant to stress factors. In this review, I present several already recognized as well as novel methods of the priming process called priming, resulting in the so-called “primed state” of the plant organism. Primed plants have a higher capability of surviving and developing under stress, and become, at least temporarily, tolerant to stress factors. In this review, several already recognized as well as novel methods of priming plants towards tolerance to metallic stress are discussed, with attention paid to similarities in priming mechanisms activated by the most versatile priming agents. This knowledge could contribute to the development of priming mixtures to counteract negative effects of multi-metallic and multi-abiotic stresses. Presentation of mechanisms is complemented with information on the genes regulated by priming towards metallic stress tolerance. Novel compounds and techniques that can be exploited in priming experiments are also summarized.

## 1. Introduction

Excessive amounts of toxic trace metal elements (heavy metals), accumulating in the soil, air, and water due to anthropogenic activities negatively affect environmental balance worldwide. In polluted land areas, toxic metals are absorbed by plants and stored in their tissues, entering the biocenotic food chains of both natural and agricultural characters. This contributes to diminished vegetable food quality because of heavy metal contamination and becomes a serious threat to human health [1]. Exposure to toxic metals itself causes growth disturbances and developmental abnormalities in the majority of plant species, namely, inhibited biomass accretion, overproduction of radicals due to impaired oxidative status, reduced photosynthetic performance, as well as changes in macromolecule synthesis and activity [2,3]. As plants are not capable of escaping stress due to their sessile form of living, they need to activate numerous defense mechanisms to counteract toxic implications of metallic stress. Some species, obligatory or facultative metallophytes, have developed particular adaptations, allowing them to grow and reproduce in heavy metal-contaminated environments [4,5,6]. Among them are hyperaccumulators capable of accumulating large amounts of toxic metals from the soil [7,8]. The whole organism responds to this environmental stress by neutralizing oxidative impairments as a result of enhanced antioxidant activity and also by phytohormonal signaling [9,10]. Metal transport to photosynthetic organs may be blocked, and then toxic ions remain stabilized in the belowground parts of the plant. At a cellular level, sequestration of toxic ions in the vacuoles occurs, accompanied by synthesis of metal-chelating compounds and ion transporters [3,11]. The ability of plants to survive and maintain proper growth under heavy metal exposure is conditioned by the collective action of these critical mechanisms. 

Defense response in the presence of metallic stress can be activated with distinct rapidity and efficiency, thus diversifying the tolerance level. Such stress defense potential can be induced though exogenously applied treatments in a process called priming, resulting in the so-called “primed state” of the plant organism. Primed plants are considered acclimated to stressful environmental conditions and are able to survive and perform better than non-primed ones. Priming has been extensively studied and applied to enhance plant tolerance to pathogen attack and to numerous abiotic stresses, predominantly drought and water deficit [12,13,14,15]. Encouraging results have provoked the opinion that plant priming towards environmental stress tolerance is among the most promising research fields in the near future [16]. 

For this reason, the aim of this compact review is to present several already recognized as well as novel methods of priming plants towards an induced acclimation to metallic stress and to critically evaluate their priming mechanisms and applicability at the both laboratory scale and in plant production practice. It was also intended to discuss future directions in this area, mainly in relation to novel compounds and techniques that can be exploited in priming experiments to enhance plant tolerance to toxic metals. Complex information on the genetic background of priming mechanisms was also gathered to facilitate the designing of future studies involving manipulations on gene expression.

## 2. What Does Priming Really Mean? The Aspects of Priming, Acclimation, and Hormesis

In studies on plant physiology, the term priming usually means short-lasting preconditioning or pretreatment with specific compounds, biological agents or physical factors in order to achieve enhanced survivability and efficient action of defense mechanisms under subsequently encountered stress [17,18]. In contrast, defense responses developed during long periods of stress duration (chronic stress, often of a gradually increasing intensity), to which plant metabolism adjusts, are attributed to the acclimation phenomenon [15,19]. Acclimated plants maintain a higher content of stress-protective compounds and therefore are well equipped for subsequent stress episodes. In this respect, priming is considered advantageous over constitutive acclimation due to its resource-saving character [15]. Priming stimulus does not modify the DNA structure permanently, and therefore its effects are reversible. Stress memory is attributed to epigenetic changes in chromatin, accumulation of inactive transcription factors, and inactive signaling compounds [20].

A primed state is defined as the physiological status in which plants are capable of quicker and more effective activating stress-protective responses [17,21]. It is a kind of “state of readiness” to another stress event, of which the duration depends on numerous factors, including the type of stress stimulus, priming agent, and physiological condition of the plant organism. Priming factors can either trigger stress itself or be a compound or stimulus that acts as a stress predictor. In this respect, priming towards metallic stress tolerance is usually achieved via trans-priming. This refers to the induction of defense response compounds other than toxic metals [20]. Most of these substances are used to stimulate plant antioxidant capacity and to counteract oxidative damage caused by heavy metal exposure [22]. Cis-priming takes place when both the priming stimulus and response triggering stimulus are of the same character, for instance, a short-term water deficit enhances a plant’s ability to survive subsequent drought [23]. In the case of toxic metal tolerance, such an approach may have confusing implications since plants subjected to transient metallic stress, particularly of low or mild intensity, exhibit a so-called hormetic effect [24,25,26]. This term refers to growth stimulation and overall better physiological performance after the application of low non-lethal doses of a toxic agent [27]. It is postulated that hormesis is an adaptive reaction to stress that allows compensating for a loss of internal balance in the poisoned organism [28]. Exploitation of intoxicated hormetic effect towards heavy metal tolerance is problematic due to environmental anxieties, and the same applies to cis-priming with trace metal ions. However, practical benefits in the form of enhanced survival during ionic stress can be obtained by priming plants with the alkali metals Na^+^ and K^+^ [29,30]. In turn, transition metal ions, particularly those regarded as essential elements, can be successfully applied as priming agents to provoke defense responses to other stresses, including drought [31,32,33], salinity [30,34,35,36], oxidative stress [37,38], as well as biotic stress [39] (Supplementary Table S1). This approach is supported by the evidence of metal involvement in cross tolerance to unfavorable conditions, even at an ecosystem level [40,41].

## 3. Priming at Critical Growth Stages 

Priming can be applied at various developmental stages of the plant life cycle and to various plant parts. The most frequently used is seed priming that serves as a germination synchronizer and has numerous advantages from an agricultural viewpoint. The germination rate of seeds soaked in water prior to sowing is usually enhanced, and seedlings grow vigorously, uniformly developing their root system [42]. These features have a significant impact on further plant growth and performance, particularly on the crop yield. Seed priming by hydration and drying was developed to increase the quality of seeds and thus to enhance their agricultural value. The simplicity of seed treatment allows wide exploitation of the technique. During soaking, seeds bind water and absorb protective and biologically active compounds. As a result, the procedure activates various enzymes, metabolic pathways, and synthesis processes that are regarded as “pre-germinative metabolism” [43,44]. Promoting effects are then manifested in developing seedlings and sustain their higher vigor and survivability under unfavorable environmental conditions. As advanced priming techniques emerged, such as nano-priming and magneto-priming, obtaining plants that are tolerant to both biotic and abiotic stresses, became even more efficient [45,46]. Moreover, storage of seeds is more convenient as there is no need for dehydration after treatment [47].

Less often, priming is applied to seedlings or their parts, as well as young plants in active growth phases. This approach allows focusing on amelioration/evaluation of defense responses in specific plant regions or organs, for instance, the root system [48,49]. In the case of metallic stress tolerance/defense, pretreatment with plant growth regulators (PGR), called hormopriming, is conducted on seedlings and young and mature plants rather than on seeds [50]. 

Priming can be applied to organs or their fragments excised from donor plants using in vitro culture. Such an approach is rather labor-consuming and requires laboratory equipment necessary to establish proliferating cultures. An inevitable advantage is the possibility of wide-scale screening of priming compounds in relation to their form or concentration, with limited factors that may interfere with the results [48,51,52,53]. Since tissue culture conditions can be regarded as a stressful environment, priming helps to induce and sustain proliferation activity, leading to efficient plant regeneration. Priming can be performed in the form of specific manipulations during culture, application of chemical compounds to culture medium or explant pretreatment prior to culture initiation, or as culture biotization with growth-promoting microorganisms [54]. The main purposes of in vitro priming is stimulation of morphogenetic responses of explants, as well as synthesis and release of biologically active compounds [55,56,57]. These goals can be obtained using priming agents of physical, chemical, and biotic natures, as summarized in Table 1. Studies considering stress response show that various priming agents are capable of reprogramming plant metabolism, by inducing oxidative bursts or regulating key genes of secondary metabolites biosynthesis. This facilitates obtaining plants resistant to pathogens via regeneration from selected cell lines [58,59,60]. Considering metallic stress, in vitro culture systems focus on developing plant tolerance to chronic exposure to trace metals, contributing to the acquisition of an acclimation status. As a result of prolonged selection in the presence of stress agents, such as salinity or toxic elements, functioning of plant cells and regenerated plantlets is modified in comparison with non-exposed ones [19,61,62]. A hypothetical “priming agent” that induces acclimation to heavy metals in such culture systems has not been identified. A recent in vitro study revealed that application of a nitric oxide (NO) donor to tobacco plants cultured in vitro in the presence of Cd or Cu ameliorated growth by elevating rubisco and rubisco activase activity in comparison with plants treated with toxic metals without NO pretreatment [63]. Notwithstanding, reports evaluating typical priming effects in in vitro culture are still scarce due to the novelty of this concept.

## 4. Major Players in Chemical Priming towards Toxic Metal(Loid) Tolerance 

Chemical priming consists of pretreatment with chemical compounds of natural or synthetic origin. Substances may be of a versatile chemical nature, from simple, purified compounds to multi-compound mixtures and extracts. The vast reports on chemical priming towards tolerance to metallic stress employ compounds involved in signaling and modulation of antioxidant response. It is a fruitful priming approach towards trace metal tolerance since enhanced activity of antioxidant machinery is crucial for compensation of oxidative imbalances caused by exposure to toxic metal ions.

Small signaling compounds (SSC), such as hydrogen peroxide (H_2_O_2_), gasotransmitters–nitric oxide (NO), and hydrogen sulfide (H_2_S), and phytohormone salicylic acid (SA) are the most frequently applied to induce priming effects in plants, regardless of the stress considered. These compounds trigger stress signals and activate defense mechanisms in a compound-specific manner; however, their common feature is the capability of inducing radical scavenging and antioxidant capacity. Moreover, all these substances interfere with each other, down- or up-regulating their quantity and metabolic pathways. Exogenously applied, the compounds influence the pool of their endogenous forms, and their orchestrated action contributes to the development of stress defense response.

Existing relationships and cross-talks between SSC have been excellently reviewed in several important papers [64,65,66,67,68,69], and interested readers can find comprehensive information. However, a detailed survey on the interactions of the four compounds taking place during heavy metal exposure is still missing. In this compact review, the results of applications of the respective compounds are presented in Table 2, with studies focusing on the interplays between at least two of them particularly highlighted. Small signaling compounds are the most versatile priming agents used for metallic stress tolerance induction, and the basics of their priming features are summarized below. Methodological details concerning pretreatments with small regulatory compounds in heavy metal-focused studies are summarized in Table 2.

*Hydrogen peroxide*. The most frequently reported is the priming effect of hydrogen peroxide (H_2_O_2_), which in fact has prooxidant properties. Low doses of H_2_O_2_ induce a primed state in plants by exposing the organism to oxidative challenge of low intensity. During pretreatment, reactive oxygen species (ROS) are generated, leading to activation of ROS-dependent signaling networks. Subsequently, accumulation of defense proteins, including transcription factors and proteins actively involved in ROS scavenging, takes place, preparing the plant for forthcoming stress events [70]. The potential of H_2_O_2_ to counteract metal toxicity was comprehensively reviewed by [71], who discussed its involvement in signal transduction by oxidizing regulatory proteins, phytohormones, and their precursors, transcripts, as well as other signaling compounds. Priming effects of hydrogen peroxide are visible at low doses, ranging from 100 to 600 µM (Table 2). At the level of plant growth and physiological status, the main benefits of H_2_O_2_ priming prior to metal treatment include decreased ROS production and oxidative damage of cell components. As a consequence, growth reduction is minimized [72,73]. The efficiency of antioxidant systems increases due to activation of the glutathione-ascorbate cycle and antioxidant enzymes [74,75]. Increased contents of glutathione are also related to improved metal sequestration via chelating and binding with phytochelatins [76,77]. Enhanced production of cysteine-rich compounds may also change the pattern of metal accumulation in plants, resulting in stabilization of toxic ions in the root system instead of their transport to photosynthetic parts [71]. Regulation of metal transport is associated with the H_2_O_2_ effect on the activity of vacuolar transmembrane proteins, such as ATPases [78]. Mechanisms of priming action of H_2_O_2_ are not limited to stimulation of the antioxidant system, but also include other cellular systems cooperating during stress. Among them is the glyoxalase system, composed of enzymes that detoxify methylglyoxal, a compound overproduced under environmental stress that is toxic at high concentrations [79]. Apart from a reduction in oxidative damage, rapeseed plants pretreated with H_2_O_2_ are capable of maintaining high activity of glyoxalases under Cd exposure. Another effect of H_2_O_2_-priming properties under metallic stress is balanced osmoregulation due to osmolyte accumulation. Enzymes of metabolic pathways of proline synthesis are upregulated after H_2_O_2_ pretreatment, whereas the activity of proline-degrading enzyme is reduced [73,75,80].

*Nitric oxide.* Nitric oxide is a hydrophobic gaseous free radical, exhibiting both prooxidant and antioxidant properties [81], and exerting either cytotoxic or cytoprotective effects in plants. Therefore, its exploitation as a priming agent depends on the dose and the mode of application of NO-donor compounds [16]. Due to its gaseous nature, NO has to be applied to plants in the form of a donor compound. In studies concerning plant reaction to metallic stress, the most frequently used NO donor is sodium nitroprusside (SNP), but other substances are exploited as well (Table 2). In an experiment on rice subjected to Cd stress, 30 µM S-nitroso-*N*-acetylpenicillamine (SNAP) functioned as a NO donor, increasing plant tolerance by improved antioxidant activity and plasma membrane structure [82]. In turn, it was found that SNP analogues: sodium nitrite and sodium ferrocyanide, unlike SNP, did not exhibit ameliorative activity under Cd stress [83]. Notwithstanding, [84] pointed out beneficial effects of NO donors such as potassium nitrite (PN), S-nitrosoglutathione (GSNO), diethylenetriamine/NO adduct (DETA/NO), SNAP, and S-nitroso-mercaptosuccinic acid (S-nitroso-MSA) on plants exposed to a wide range of environmental stresses.

Nitric oxide belongs to gasotransmitters—small molecules involved in plant physiology regulation [85]. Because of its versatile signaling role, NO is widely applied as a priming agent to enhance plant survival under abiotic stresses. [86] provided a comprehensive review of NO effects on plants treated with cadmium. They proposed that mechanisms of NO priming action involve protein modifications, activation of secondary messengers, including phytohormones and ROS, as well as alteration of protein kinase activities [86]. Survey of recent literature showed that the priming effect of NO and its interactions with other SSC are abundant in plants exposed to metallic (metalloid) stress, regardless of the toxic metal type [68,87,88,89] (Table 2). NO ameliorates plant growth as a result of reduced ROS production, minimization of oxidative damage, and improved antioxidant capacity [83,90,91,92]. Redox balance of the cell is maintained by an alternative redox-regulation system, composed of methionine sulfoxide reductases (MSR) [18]. Owing to the MSR capability of maintaining a proper redox state of methionine and preventing protein oxidation under environmental challenge, larger pools of glutathione and chaperones may be available for counteracting other stress effects in the cells [93]. Recent reports indicate that NO priming stabilizes redox status in plants exposed to toxic metals; thus, enhanced expression of MSR genes can be avoided [18]. In comparison with non-primed plants, NO-primed ones have a modified structure of cell walls and activity membrane transporters, and thus transportation of toxic ions is altered [68,82,94]. Photosynthetic performance is also improved, contributing to enhanced biomass accretion and proper organ development [63,87].

*Hydrogen sulfide.* Similar to NO, hydrogen sulfide (H_2_S) is a gasotransmitter that can be either beneficial or toxic to plants. In millimolar concentrations it was considered phytotoxin for ages; however, in recent years its signaling role in plant organisms was ascertained. Studies revealed that the endogenous molecule is involved in numerous physiological processes, and a set of enzymes is available to regulate H_2_S internal concentrations [95]. This compound interacts with other signaling molecules, mainly phytohormones and gasotransmitters, influences the functioning of ion channels and miRNA (micro RNA), thus indirectly regulating cell membrane polarization and transcription [67]. Priming action towards metallic stress is mainly attributed to enhanced antioxidant activity, particularly of the enzymes belonging to the AsA-GSH cycle [96], which contributes to reduced accumulation of ROS [89], improved metal chelation by GSH and phytochelatins [97,98], and diminished oxidative damage of macromolecules [99,100]. It also reduces nitroso-oxidative stress by interfering with NO- and ROS-associated metabolism [101]. H_2_S interactions with H_2_O_2_ and NO are particularly reflected in changes in the activity of membrane transporters, affecting metal(loid) uptake and translocation [68,78]. Exogenous H_2_S is applied in the form of donor compounds that degrade to release H_2_S in the cells [102]. In plant systems, donor sulfide salts, mainly sodium hydrosulfide (NaHS) and sodium sulfide (Na_2_S), are usually used (Table 2). Their solutions are easy to prepare and apply; however, release of gaseous H_2_S to the atmosphere is quite fast. Recently, slow-release donor GYY-4137 (P-(4-methoxyphenyl)-P-4-morpholinyl-phosphinodithioic acid), which releases H_2_S as a result of hydrolysis (and probably also in an enzymatic way) in a prolonged time period, was found to be more convenient to handle and experiment on plants [103]. There are no reports on its effect during metallic stress, but these are expected in the near future. An emerging area is the development of novel H_2_S donors that are particularly important for medical purposes [102,104]. As these new drug formulations are developed, some of them may be applied in plant systems as prospective priming compounds (see the subsections below). The most important for broad agricultural exploitation is the low cost of a donor compound and the ease of simultaneous treatment of numerous seeds/plants [69].

*Salicylic acid*. Salicylic acid belongs to phenolic acids and is considered a plant growth regulator; thus, SA pretreatment is classified as hormopriming. Its mechanism of signaling and regulatory action, also involved in priming effects, is related predominantly to an interplay with a pool of other phytohormones and endogenous H_2_O_2_. SA is applied to prime plants against biotic stresses as it triggers the immune response to counteract pathogen attack [48] and against abiotic environmental stresses since it takes part in response development under osmotic and oxidative stress [105]. These “secondary stresses” are associated with salinity, ozone treatment, an exposure to toxic metals as well as pathogen attack [48,106]. Considering metallic stress, successful priming with SA was reported for several metals and in numerous plant species (Table 2). Beneficial effects were manifested mainly in an increased antioxidant capacity to scavenge ROS and other radicals [107,108,109,110] and in inhibited accumulation of toxic elements [98,107,109,111,112]. Pretreatment with SA prior to metallic stress affects the content and rearranges the profile of phenolic and organic acids [112], amino acids [98], as well as lipids and fatty acids [113,114]. A study on *L. usitatissimum* showed that the composition of plastidial membranes was preserved due to SA application prior to Cd treatment, which suggests the protection of the photosynthesis system from damage [113].

In light of recent findings, it seems that the plant capability of maintaining a relatively low content of endogenous SA and avoiding its elevation contribute to enhanced tolerance to metallic stress [115,116]. It is unresolved whether SA priming affects the biosynthesis pathway of endogenous SA and its final content. From a more practical point of view, studies evaluating the priming effect of SA derivatives and analogs, i.e., o-acetylosalicylic acid or 2,6-dichloroisonicotinic acid, are missing. These compounds enhance the plant response to other stresses, such as drought and salinity [14,106]; thus, it is substantiated to verify their suitability to induce trace metal tolerance as well.

From this compact review perspective, the similarities in response triggering may be used to elaborate a formulation for a “priming cocktail” that can be easily and at low cost applied in croplands to induce stress and even multistress tolerance. Regardless of their sole or joint application, small signaling compounds significantly contribute to increased survival and growth of plants in the presence of metallic elements.

## 5. Other Potent Chemical Priming Agents

*Polyamines*. Polyamines (PAs) are polycationic compounds with two or more amino groups. They occur ubiquitously in living organisms, and the most abundant are diamine putrescine (Put), triamine spermidine (Spd), and tetraamine spermine (Spm) [117]. These active PAs participate in multiple biological processes, from the cellular to the whole organism level, having regulating and signaling functions [118]. In plants under stress, their ameliorative role is attributed to support the radical scavenging system and antioxidant response, modulation of cation channels and membrane stabilization, regulation of phytohormone synthesis, and epigenetic modifications [119]. From the priming point of view, particularly interesting are relationships between polyamines and SSC. During PA catabolism, production of H_2_O_2_ occurs, and the pool of SA is affected [120]. Additionally, PAs are substrates for NO synthesis [85]. Exogenous treatment with PAs may therefore alter the levels of these SSC and contribute to intensified tolerance response to metallic stress. PAs also form conjugates with hydroxycinnamic acid that structurally resemble phytochelatins and are likely involved in metal chelation [121]. In priming experiments aimed at inducing metallic stress tolerance, usually micro-millimolar (0.1–1 mM) solutions of Put, Spr, and Spm are used for soaking seeds or for foliar spraying. PAs stimulate the antioxidant system and reduce metal accumulation and macromolecule damage, as shown in studies on plants stressed with Cd, Pb [122], Cr [123], and Co [124].

*Melatonin and its derivatives*. The role of indoleamine melatonin (*N*-acetyl-5-methoxytryptamine) (Mel), an animal hormone, in plant functioning was repeatedly reported in recent years [125,126] when improved detection methods allowed precise determination of its content in plant tissues. It became apparent that both Mel and its metabolite, 2-hydroxymelatonin (2-OHMel), are capable of inducing tolerance responses in stress-exposed plants and therefore are now exploited as chemical priming agents for chilling, salinity, and drought [127,128]. They are also efficient priming agents for combating metallic stress. Ameliorative action under metal exposure is mainly attributed to the modulation of antioxidant responses, enhanced accumulation of phenolics, carbohydrates, polyamines, and proteins, as well as to restricted metal translocation [129,130,131,132,133]. It has been recently shown that Mel protects cells from DNA damage and subsequent cell death induced by Pb [51].

*Beneficial elements.* Another strategy to increase toxic metal tolerance is the application of beneficial elements as priming agents, sometimes referred to as nutripriming. Solutions of numerous compounds are usually inexpensive and easy to handle and therefore, large amounts of seeds/seedlings can be rapidly primed. Recent reports show that encouraging results have been obtained in plant priming with silicon (Si) and selenium (Se) compounds. Si, applied as 10^−5^ M sodium metasilicate, ameliorated growth and the physiological response of *Brassica juncea* under Ni exposure by stimulation of antioxidant machinery [134]. In *Capsicum annuum* subjected to Cd stress, Si applied as 2 mM sodium silicate, apart from stimulation of antioxidant system activity, promoted the accumulation of endogenous H_2_O_2_ and NO [135]. Enhanced synthesis of another SSC, H_2_S, occurred in zucchini plants primed with Ca^+2^ ions prior to Ni exposure [97]. Rice seedlings primed with 60 µM Se had a more efficient antioxidant system and performed better under nutrient imbalances caused by Pb and Ni [107,108]. Moreover, translocation of toxic ions can be significantly restricted as a result of Se priming [136]. Inevitably, an enormous potential for elemental priming against metallic stress is represented by nanoparticles. Their application causes significant improvements in antioxidant responses under metallic stress, as in the case of sunflower seeds primed with S nanoparticles prior to Mn treatment [137]. Se nanoparticles also have potential, as foliar spraying with this form of Se facilitated the synthesis of non-enzymatic antioxidants and enhanced the activity of antioxidant enzymes in coriander plants exposed to Pb stress [138]. A recent study revealed that Se nanoparticle application influenced not only the antioxidant system but also altered plant morphology and metabolite synthesis and contributed to the accumulation of epigenetic changes in DNA [139]. Such a versatile modulation of plant metabolism may facilitate the development of metallic stress tolerance as well.

## 6. Genes That Can Be Targeted to Induce Priming Effects for Metallic Stress

Genetic control of the priming phenomenon involves the regulation of numerous pathways activated during pre-treatment and subsequent stress events. New information on its mechanisms are gathered due to “omics” analyses that decipher changes in the transcriptome, proteome, and metabolome of primed plants [44,140,141,142]. However, the details of genetic control during priming against metallic stress are not fully elucidated. The recognition of potential genes that can be targeted may facilitate future studies on priming effectiveness towards tolerance to metal toxicity. Recent studies confirm that priming agents are potent activators of the genetic regulatory network and contribute to diversified expression of genes encoding stress-defense compounds in plants cultivated in contaminated soil [44]. The genes can be classified as those that control development and common versatile defense responses in plants, irrespective of the stress type and genes that are specifically expressed in the presence of toxic metals. The first group would include genes encoding (i) enzymes involved in antioxidant response pathways— the whole set of radical scavenging enzymes, including proteins of the ascorbate–glutathione cycle, as well as the enzymes of supportive redox systems (glyoxalase system, methionine sulfoxide reductases) [18,79], (ii) proteins involved in SSC production, (iii) enzymes crucial for PA and Mel biosynthesis pathways [143,144], (iv) heat shock proteins and other chaperones responsible for proper folding and (de)aggregation of proteins [145], (v) DNA repair enzymes [44], and (vi) proteins regulating developmental processes, such as stress-responsive asparagine rich protein (NRP) [146], signal transducing mitogen-activated kinases (MPK) or proteins involved in chromatin modifications [141]. As depicted in the subsections above and in Table 2, the priming agents predominantly affect the antioxidant activity, and numerous studies confirmed that enhanced expression of genes encoding antioxidant enzymes, superoxide dismutase (SOD), peroxidases (POD), and catalase (CAT), enzymes of the ascorbate–glutathione cycle, occurs as a priming effect [44,75,88]. Additionally, SOD and, in the presence of a strong reductant, also peroxidases take part in H_2_O_2_ production in numerous cell compartments [71]. Among the genes encoding enzymatic machinery for the synthesis of other SSC, interest should be paid to cysteine desulfhydrolase (CAS) (catalyzing H_2_S formation) [68], the large gene family of nitric oxide synthases (NOS) (catalyzing NO synthesis) [147], as well as isochorismate synthase (ICS) and phenylalanine ammonia-lyase (PAL) involved in SA biosynthesis [148].

Putrescine and spermine are PAs particularly involved in stress tolerance (Put) and stress response (Spm) [117]. There is evidence that under stress, biosynthesis of endogenous Put occurs more frequently via an indirect route from arginine (in the arginine decarboxylase pathway ADC) rather than directly from ornithine (in the ornithine decarboxylase pathway ODC) [121,149], which suggests that the genes encoding proteins active in the ADC pathway should be chosen as prospective modification targets. In order to increase Spm accumulation, the activity/expression of the key rate-limiting enzyme, S-adenosylmethionine decarboxylase (SAMD), could be manipulated [143]. In the case of Mel, its biosynthesis may be promoted when a gene encoding the enzyme serotonin N-acetyltransferase (SNAT) is overexpressed [144].

Plant cells are equipped with numerous stress- and damage-protective proteins whose expression and functioning can be manipulated to achieve priming effects. Heat shock proteins (HSPs) are involved in the response to environmental factors in almost all living organisms. Genes encoding plant HSPs are localized in nuclear and cytoplasmatic genomes, whereas proteins are abundant in various cell compartments. Diverse HSP variants detected in plants probably reflect molecular modifications forced by various stress conditions [150]. Although HSPs function in a wide range of abiotic stresses, including oxidative stress, the understanding of their specific roles in plants cells is still limited [151]. The involvement of mitochondrial sHSP23 and cytosolic sHSP17.7 proteins in As (as a metalloid stress) and Pb tolerance has been reported in plants and bacteria [152,153,154], whereas expression of maize root HSP70 was upregulated by cesium oxide nanoparticles [155].

Protective functions also include proteins responsible for DNA repair since genome stability is essential to maintain growth and development [156]. The enzymes 8-oxoguanine glycosylase (OGG1) and formamidopyrimidine-DNA glycosylase (FPG) act through the base excision repair pathway, cutting off DNA bases that were irreversibly oxidized during stress exposure. Expression of genes encoding OGG1 and FPG was upregulated by hydro- and biopriming, applied to improve plant performance during germination and growth in metal-contaminated soil [44]. It is noteworthy that the expression of DNA repair enzymes is particularly critical during seed imbibition and germination [156], the predominant stages in which priming is applied.

To the second group belong genes encoding (i) metal transporters, particularly vacuolar, such as ABC (ATP-binding cassette) proteins [78], CDF (cation diffusion facilitator), but also cytoplasmatic (ZIP–zinc/iron transport proteins: ZRT/IRT-related proteins) [157]. Included here are also (ii) genes encoding metal-binding peptides: metallothioneins (MTs) and enzymes responsible for the synthesis and transport of metal chelators: phytochelatins and low-molecular-weight organic acids (LMWOA). Phytochelatin synthase (PCS) is considered a crucial enzyme for metal tolerance and plant capability of phytoremediation [158]. In the case of LMWOA, the main role in acquiring metal(loid) tolerance is played by organic acid anion transporters belonging to the MATE (multidrug and toxic compound extrusion) family [68]. Products of their expression actively take part in metal detoxification by restricting or facilitating their translocation between plant organs and by exclusion of toxic ions from the cytoplasm and their sequestration in the vacuole. Additionally, MTs may function as DNA-repair compounds and therefore, their priming-related action may be more versatile [43]. Expression of MT-2 genes is particularly influenced during cell response to oxidative stress [44].

## 7. Hydropriming, Halopriming, Hormopriming, and Biopriming

These priming strategies involve the application of water, salt solution, phytohormones, and promoting microorganisms as priming agents. Suitability of *hormopriming* to achieve metallic stress tolerance and the protective mechanisms of particular phytohormones were recently comprehensively discussed in [50]. Here, this aspect will not be extensively presented since the mechanisms of action and impact of salicylic acid, the most frequently used priming agent among growth regulators, were described in the subsection above. However, all types of phytohormones, both growth promoters (auxins AUX, cytokinins CYT, gibberellins GAs) and inhibitors/regulators (abscisic acid ABA, jasmonic acid JA), as well as brassinosteroids (BR) and strigolactones (SL) are potent priming agents. On a molecular level, their stress-preventing action is attributed to signaling via phytohormonal cross-talks and interactions with the SSC synthesis pathways [49], regulation of gene expression, and influencing post-transcriptional protein modifications, as well as epigenetic control of stress-related genes [159,160]. Phytohormones are applied to plants via seed soaking, foliar spraying, and medium enrichment, with micromolar concentrations exerting a positive impact on plant functioning under metal exposure. Besides SA application, priming with AUX is the most frequent approach in combating metallic stress. Indole-3-acetic acid (IAA) and indole-3-butyric acid (IBA), applied in concentrations ranging from 2–500 µM, ameliorate the toxicity of cadmium [111,161,162,163,164,165], copper [124,166,167], and aluminum [168]. Numerous studies report the beneficial activity of low BR doses (0.5–10 µM) (24-epibrassinolide, 3-epibrassinolide) towards stress induced by metals and metalloids: As, Cd, Cr, and Cu [109,123,169,170]. GAs (1–100 µM) are effective priming agents diminishing the growth deteriorations caused by Cu and Zn [167,171]. CYT (i.e., 10–100 µM 6-benzylaminopurine) reduced the detrimental effects of Cd and Zn toxicity [172,173], whereas jasmonic acid (0.1 µM JA)– ameliorated reaction to Pb stress [174]. Recently 1.0 µM SL analogue GR24 was found to combat Cd toxicity in switchgrass (*Panicum virgatum*) by altering nutrient and ballast metal uptake and interacting with the pool of endogenous strigolactones [175].

*Halopriming* involves salt treatments applied prior to exposure to stress conditions. It is commonly used in the form of cis-priming to induce tolerance to salinity and other osmotic stresses [176]. Recently, it was revealed that 10-h priming of wheat seeds with 7.5 mM Mg(NO_3_)_2_ and Ca(NO_3_)_2_) improved seed germination under Hg application by stimulation of growth of seedling organs and carbohydrate metabolism [177]. Similar effectswere achieved by *hydropriming*, which consists of seed soaking in distilled water, without any other components [177]. An inevitable advantage of hydropriming is its simplicity and low cost, as well as the possibility to achieve a large population of primed plants. It has been long applied in agricultural production to synchronize seed germination and to improve yield [46]. Apart from improving germination characteristics, hydropriming also affects the expression of genes involved in antioxidant defense and metal chelation, as revealed in a *Medicago truncatula* grown in contaminated soil [44]. However, in numerous studies analyzing priming effects towards metallic stress tolerance, hydropriming is regarded as a control and as a reference treatment for proper assessment of the impact of priming compounds suspended in water solutions.

*Biological priming***,** namely, priming by colonization of plants with beneficial microorganisms to induce stress tolerance, is gaining interest worldwide since it fits in and complements sustainable and organic plant cultivation. Beneficial microorganisms can be isolated and selected from heavy metal-polluted soils, particularly from the rhizosphere of metal-resistant plants, but also from aboveground organs. Bacterial species with plant growth-promoting activity belong to numerous genera, i.e., *Alcaligenes*, *Curtobacterium*, *Bacillus*, *Microbacterium*, *Paenibacillus*, *Pseudomonas*, *Streptomyces,* and *Tetrathiobacter* [178,179,180], whereas among fungi are rhizosphere endophytes, such as *Piriformospora indica, Acrocalymma vagum,* and *Scytalidium lignicola* [181,182]. Improved plant growth under metallic stress is attributed to bacterial capability of nitrogen assimilation, producing and secreting plant growth regulators, particularly IAA, siderophores, and various volatile organic compounds [183,184,185]. Fungal species stimulate the antioxidant system in plants and prevent cell death, as well as ameliorate water uptake, facilitating access to less available nutrient elements. Toxic metals can be solubilized or adsorbed on microorganism biomass, restricting their bioavailability and uptake by plants [186]. At the regulatory level, biopriming interferes with the expression of genes involved in stress responses [44,187], as well as with phytohormonal regulatory network [184].

## 8. Effect-Oriented Priming: Growth Improvement, Metal Uptake Restriction or Metal Uptake Stimulation?

**Figure 1:**
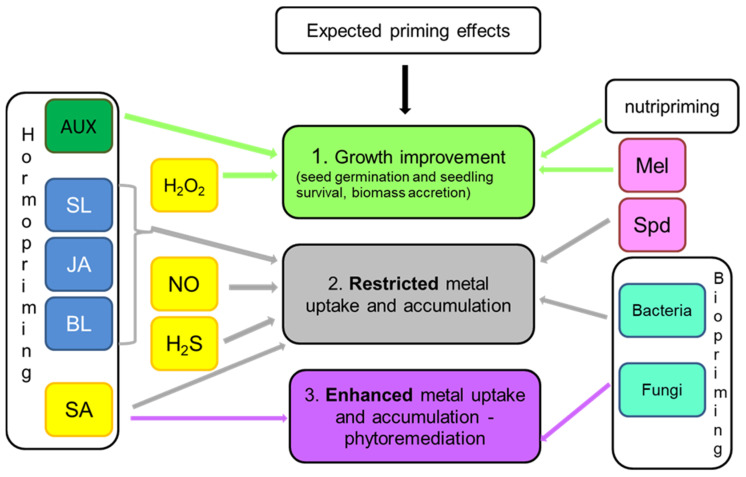
Effect-oriented priming—the choice of priming agent depends on the expected outcome of the priming procedure.Small signaling compounds (SSC) were colored yellow. Abbreviations: AUX—auxins, BL—brassinosteroids, JA—jasmonic acid, Mel—melatonin, SA—salicylic acid, SL—strigolactones, Spd—spermidine.

Considering plant cultivation under exposure of metallic elements, the desired goals of priming can be directed either to improve seed germination, survival, and biomass accretion or to reduce uptake and transport of toxic ions in plant organs. Obviously, all priming agents are applied to achieve improved plant growth and yield, but some of them are also particularly effective in restricting metal accumulation. Analysis of available reports shows that the highest potential for decreasing metal accumulation in plant organs has chemical priming with the use of NO and H_2_S donors, as well as with triamine–spermidine (Figure 1). NO and H_2_S affect plasma membrane proteins, mainly transporters and antiporters [68,78,82], whereas spermidine and its complexes take part in metal chelation and influence metal transportation within plant tissues [122,123]. The metal content in biomass can be also reduced by priming with PGRs with growth-inhibiting properties: JA, brassinosteroids, and strigolactones [169,174,175]. Among other techniques, the use of biopriming with selected bacterial bioremediator strains allows achieving a significant decrease in toxic metals accumulated in the plant yield [179,183,185]. There are also reports indicating that nutripriming with Se is effective in reducing the as content in the grains and thus restricts its circulation in the food chain [136].

Priming techniques aimed at improving plant growth and physiological performance involve priming with H_2_O_2_ that stimulates antioxidant defense [75,79,80] and hormopriming with PGRs of growth-promoting activities, particularly with AUX [162,163,164]. Application of melatonin, which is an indole compound sharing some routes of the biosynthetic pathway with AUX, also facilitates plant survival and biomass accretion under metallic stress [131,132,133] (Figure 1).

Another option is to use priming agents for stimulating metal removal from contaminated soil, facilitating its clean-up by phytoremediation. In such a case, a priming compound is expected to enhance metal uptake and accumulation by plants. Such effects can be obtained by priming with SA since this molecule differentially influences metal distribution between roots and shoots [107,112] (Figure 1). This feature can be used to manipulate a plant’s ability to phytostabilize (in the root system) or phytoextract (in the aboveground biomass) metals. Significant improvement in metal uptake from the soil is also obtained by biopriming with fungal endophytes [182].

## 9. Prospective Priming Approaches and Concluding Remarks

Chemical priming gives an opportunity to elaborate “priming cocktails”, mixtures of priming agents of which joint action would contribute to further enhancement of plant growth and productivity under unfavorable conditions. A multiple priming approach seems crucial if negative effects of multistress, encountered by plants under natural conditions, are to be diminished. Application of priming cocktails may also facilitate organic and sustainable crop production and reduce the amounts of agrochemicals [16]. A successful and prospective example of a versatile priming agent is NOSH-aspirin, a compound that acts as a donor of NO, H_2_S, and the SA derivative acetylosalicylic acid. Priming with NOSH-aspirin ameliorates the drought stress reaction of alfalfa plants by inducing typical defense reactions, particularly antioxidant capacity [14]. This novel compound is currently tested by patent owners in other plant-stress systems, including heavy metal exposure (communicated by Prof. K. Kashfi).

To combat metallic stress via priming, new priming agents and techniques are constantly developed and incorporated into practice. Some prospective multiple priming agents can be found among compounds applied in the case of other stresses. For instance, apoplastic fructans, flavonoids or amino acids (i.e., β-aminobutyric acid, vitamin U– S-methylmethionine) are capable of priming plant innate immunity and increasing cold tolerance [12,188,189], but their stress-protective properties can be verified under metallic stress as well. One prospective is the exploitation of nanoparticles that induce significant variations in plant biochemistry, gene expression, and epigenetic DNA patterns [139], contributing to the development of altered (and probably more efficient) stress responses. Among physical priming methods, magnetopriming was reported to stimulate antioxidant system activity under salt stress [190], and therefore this technique may have significant potential for metallic stress amelioration. Finally, natural organic extracts can also be sources of priming agents or constitute priming cocktails themselves, as shown by a recent study on the amelioration of Pb toxicity by seed priming with weed extracts [191].

In conclusion, all priming strategies towards improved plant tolerance to toxic metal and metalloids have important features in common: Are easy to apply to numerous plants, are not labor-consuming, do not require sophisticated laboratory equipment, and are relatively inexpensive. For these reasons, priming can be routinely applied in agricultural and horticultural practice all over the world. These are inevitable advantages over stress tolerance induction via modern genetic engineering techniques, and therefore novel and improved priming methods are expected to constantly develop. The joint knowledge gathered here, covering the biochemical and genetic background of the priming phenomenon, may facilitate designing and conducting future research for plant tolerance to metal and metalloid stress.

## Figures and Tables

**Table 1:** Examples of in vitro culture priming towards plant improvement and stress tolerance.

Priming Agent	Plant Species	Culture Type	Priming Benefits	Reference
6.3 µM chlorocholine chloride	*Eclipta alba*	Shoot culture	↑ root number and length ↑biomass of regenerated plantlets ↑ chlorophyll content ↑survival after acclimatization to ex vitro conditions	[56]
1 mg·mL^−1^ ulvan (sulphated polysaccharide from green macroalga *Ulva fasciata*)	*Triticum aestivum* *Oryza sativa* *Hordeum vulgare*	Suspension culture	prevention from oxidative burst caused by chitosan ↓symptoms of infection with pathogen *Blumeria graminis*	[58]
Imprimatins	*Arabidopsis thaliana*	Suspension culture	suppressed growth of bacterial pathogens ↑level of endogenous SA ↑expression of PR genes ↓reduced SA inactivation by glucosylation	[48]
Pathogen-derived compounds (1 mM isonitroacetophenon, 300 µM acibenzolar-S-methyl, 200 nM flagellin, 100 µg·mL^−1^ chitosan, 100 µg·mL^−1^ lipopolysaccharide)	*Nicotiana tabacum*	Suspension culture	↑synthesis of mono- and di-acetylated chlorogenic acids	[59]
Ultraviolet C (UVC)	*Coleus forskohlii*	Callus culture	callus organogenesis ↑resistance to Macrophomina root rot disease (*M. phaseolina* pathogen) ↑forskolin synthesis via upregulation of forskolin biosynthesis genes	[60]
0.05 mM SNP (nitric oxide donor)	*Nicotiana tabacum*	Shoot culture	↑tolerance to Cd and Cu, manifested by higher biomass, chlorophyll content and rubisco activity	[63]
200 nM melatonin	*Nicotiana tabacum*	Suspension culture	↑cell survival in the presence of Pb	[51]
Volatile compounds released by *Microbacterium* strain EC8	*Arabidopsis thaliana* *Lactuca sativa* *Solanum lycopersicum*	In vitro-grown seedlings	↑growth promotion-shoot and root biomass, lateral root density	[52]
Cold plasma 5–80 mg·L^−1^SiO_2_ nanoparticles (nSi)	*Astragalus fridae*	Seeds germinating in vitro	↑root system development ↑ shoot biomass ↑chlorophyll and carotenoid contents ↑ root NR activity, shoot CAT activity modified anatomy and tissue differentiation patterns interactive effect of cold plasma and nSi	[53]

↓ decrease/reduction; ↑ increase/enhancement. Abbreviations: CAT—catalase, NR—nitrate reductase, PRs—pathogen related genes, SA—salicylic acid, SNP—sodium nitroprusside

**Table 2:** Procedures and effects of plant priming against metallic stress using small signaling compounds: hydrogen peroxide (H_2_O_2_), nitric oxide (NO), hydrogen sulfide (H_2_S), and salicylic acid (SA).

Metallic/Metalloid Stress	Priming Details			Species	Priming Effects in Comparison with Non-Primed Plants				References
	Dose (mM)	Duration	Plant Part		ROS Production/ Oxidation Activity	Antioxidant Response	Photosynthetic Performance/Biomass Accretion/Growth	Other	
**Hydrogen peroxide H_2_O_2_**									
As, (20–50 µM)	0.05 H_2_O_2_			*Oryza sativa*		↑ SOD, APX, GR, GSH	↑	↑ expression of genes encoding photosystem proteins and antioxidant enzymes ↑ proline	[75]
Cd (10–150 µM CdCl_2_)	5 H_2_O_2_	24 h	roots	*Cucumis sativus*			↔	↑ endogenous H_2_S ↓ ATP hydrolysis and proton transport ↑expression of genes encoding V-ATPase subunits	[78]
Cu, (10–100 mg kg^−1^ soil)	0.1–0.5 H_2_O_2_	4 h	roots	*Solanum lycopersicum*	↓O_2_·^−^, MDA	↑ CAT, POD, SOD	↑	↑ proline	[73]
Cd (0.5–1.0 mM CdCl_2_)	0.05 H_2_O_2_	24 h	roots of seedlings	*Brassica napus*	↓ H_2_O_2_, O_2_·^−^, ↓LOX	↑ AsA, GSH, APX, DHAR, GR, GST, CAT, GPX ↓DHA		↑ glyoxalase I and II activity	[79]
Ni (200 mg kg^−1^ soil NiCl_2_)	0.05 H_2_O_2_	15 days	seedlings	*Brassica juncea*	↓TBARS	↑APX, GR, GSH ↓GSSG	↑rubisco, PSII activity, leaf area	↑N and S assimilation (enzyme activity)	[72]
Cu (50 µM CuCl_2_)	0.3 H_2_O_2_	6–8 h	seedlings	*Zea mays*			↑	↑gene expression and activities of enzymes involved in proline synthesis (GDH, P5CS, arginase, OAT) ↑ proline ↓ activity of proline degrading enzyme ProDH	[80]
Cr(VI) (50 µM)	0.2 H_2_O_2_	24 h	seedlings	*Brassica napus*	↓MDA	↑NPT, PT, APX, POD	↑	↑ Cr translocation to shoots ↑ expression of metallothionein gene	[77]
Cd (50 µM)	0.1 H_2_O_2_		3-leafed seedlings	*Oryza sativa*	↔ MDA	↑GSH, NPT, PCs, GST	↑	↓ Cd translocation to shoots	[76]
Al (30 µM)	0.6 H_2_O_2_	2 h	root tips	*Triticum aestivum*	↓ H_2_O_2_, O_2_·^-^	↑ SOD, CAT, POD, APX, MDHAR, GPX, GR, GSH, AsA	↓ root elongation		[74]
**Nitric oxide NO**									
Cd (0.1 mM CdCl_2_)	0.1 SNP	28 days	seedlings	*Triticum aestivum*	↓ H_2_O_2_, MDA	↑ SOD, CAT, POD	↑	↓ proline ↓ Cd uptake and accumulation ↑endogenous H_2_S ↑ Zn^2+^, Fe^2+^, Ca^2+^, K^+^ content	[89]
Cd/Cu (0.2 mM each)	0.05 SNP	35 days	in vitro grown plants	*Nicotiana tabacum*			↑rubisco and rubisco activase content and activity, depending on the metal		[63]
Cd (10 µM CdCl_2_)	0.03 SNAP	2 h	3-leafed seedlings	*Oryza sativa*	↓ H_2_O_2_	↑GSH, APX, SOD, GR	↑	affected abundance of plasma membrane proteins (transporters, ATPases, kinases, phosphatases, phospholipases, enzymes, antiporters, structural proteins, aquaporins, signal, and hormone-related proteins) ↑phosphatidic acid	[82]
Cd (100–200 µM CdCl_2_)	0.25 SNP	14 days	seedlings	*Arachis* *hypogaea*	↓O_2_·^−^, MDA	↑ SOD, CAT, POD, AsA	↑	↓ Cd translocation to shoots, ↑ enhanced Cd binding in cell walls ↑ proline	[92]
Mn (1000 µM MnCl_2_)	0.1–1 SNP	7 days	seeds	*Matricaria chamomilla*	↓ ROS	↑ APX ↓ CAT	↑	↓ Mn content in roots and shoots	[91]
Al (100 µM AlCl_3_)	0.01–0.05SNP	12 h	seedlings	*Glycine max*				cooperates with H_2_S in induction of citrate transporter expression ↑ endogenous H_2_S ↑ activities of H_2_S biosynthesis enzymes (cysteine desulfhydrases, CAS) ↓activity of H_2_S-degrading enzyme (OAS-TL)	[68]
Pb (50 µM Pb(NO_3_)_2_)	0.1 SNP	2–8 h	germinating seeds	*Triticum aestivum*	↓MDA, conjugated dienes, O_2_·^−^, ·HO	↑ APX, GPX, GR, SOD	↑ radicle and plumule length	↑intracellular nitrite content	[87]
Cd (100 µM)	0.05 SNP			*Trifolium repens*	↓ H_2_O_2_, MDA	↑ ROS scavengers (enzymatic and non-enzymatic)	↑	↓ inhibition of H^+^-ATPase proton pumps ↑ jasmonic acid, proline ↓ salicylic acid, ethylene ↑ Mg^2+^, Cu^2+^, Ca^2+^, Fe^2+^	[83]
Ni	SNP			*Oryza sativa*	↓ H_2_O_2_, MDA	↑AsA, POD, CAT	↑	↑soluble proteins↑ proline↑ transcript levels of CAT, POD, APX, GR, SOD genes	[88]
As (0.25–0.5 mM NaHAsO_4_)	0.25 SNP	72 h	seedlings	*Triticum aestivum*	↓ H_2_O_2_, MDA	↑AsA, GSH, GSH/GSSG, MDHAR, DHAR, GR, GPX, CAT ↓GSSG		↑ proline ↑ glyoxalase I and II activity	[90]
Cd (100 µM CdCl_2_)	0.1 SNP	3 days	plants	*Arabidopsis thaliana*	↓protein oxidation ↓ROS/peroxides	↑GPX, APX, CAT		↓expression of methionine sulfoxide reductase family genes	[18]
**Hydrogen sulfide H_2_S**									
Cd (0.1 mM CdCl_2_)	0.2 NaHS	28 days	seedlings	*Triticum aestivum*	↓ H_2_O_2_, MDA,	↑ SOD, CAT, POD	↑	↓ proline ↓ Cd content ↑endogenous NO ↑ Zn^2+^, Fe^2+^, Ca^2+^, K^+^ contents	[89]
Al (100 µM AlCl_3_)	0.01–0.1 NaHS	12 h	seedlings	*Glycine max*			↑ root growth	↓ Al content in root tips ↑ citrate secretion upregulation of plasma membrane H+-ATPase (proton pump) act downstream of NO activity in Al-tolerance	[68]
Cd (5–50 µM CdCl_2_)	0.2 NaHS	48 h	seedlings	*Hordeum vulgare*	↓ MDA,H_2_O_2_, O_2_·^-^	↑SOD, POD, APX ↑GSH, AsA (at moderate Cd level) ↓CAT	↑	↓ Cd content	[100]
Cd (10–150 µM CdCl_2_)	0.1 NaHS	24 h	roots	*Cucumis sativus*			↑	↑ endogenous H_2_S ↔ endogenous H_2_O_2_ ↑ ATP hydrolysis and proton transport ↑expression of genes encoding V-ATPase subunits ↑activity and transcripts of plasma membrane NADPH oxidase	[78]
Pb (100–400 µM Pb(NO_3_)_2_)	0.1–0.2 NaHS	15 d	seedlings	*Brassica napus*	↓ MDA, H_2_O_2_, O_2_·^−^, -OH	↑SOD, POD, APX, CAT, GR, AsA, GSH, GSSG	↑biomass	↓ Na^+^ uptake ↑micro- and macroelement uptake ↑ total soluble proteins	[99]
Pb (2.5 mM Pb(NO_3_)_2_)	0.2–2 NaHS	12 h	seeds	*Zea mays*		↑ GSH	↑	↓ Pb content ↓ amino acids: Asp, Glu, Asn, Ser, Hist, Gly, Threo, Ala, Cyst ↑amino acids: Tyr, Tryp ↑NR activity ↑protein content	[98]
Cr (200 µM K_2_Cr_2_O_7_)	0.5	9 d	seeds	*Zea mays*	↓ H_2_O_2_	↔GPOX ↓GST, SOD, GSNOR	↑ radicle length	↓protein carbonylation and thiol oxidation ↑endogenous NO ↓NADPH oxidase activity ↑S-nitrosoglutathione	[101]
**Salicylic acid (SA)**									
Cd (0.25–0.5 mM Cd(NO_3_)_2_)	0.5 SA	24 h	seeds	*Silenesendtneri*		↑POD	↑germination and seedling development (root length, shoot biomass)	↑ Cd content in shoots ↓ Cd content in roots altered profile of secondary metabolites: phenolic compounds and organic acids	[112]
Pb (1 mM PbCl_2_)	100 mg·L^−1^ SA	24 h	seeds	*Oryza sativa*	↓ H_2_O_2_, O_2_·^−^, −OH, MDA	↑ SOD, POD, CAT ↑GSH	↑ shoot growth	↓Pb content in shoots ↔ Pb content in roots ↔ macronutrient uptake	[107]
Ni (0.25 mM NiSO_4_	100 mg·L^−1^ SA	24 h	seeds	*Oryza sativa*	↓ H_2_O_2_, O_2_·^−^, -OH, MDA ↓XOD, MAO	↑ CAT, GR, SOD, GPX, POD ↑GSH, AsA, Ve	↑ shoot growth	↑uptake of macronutrients ↓Ni content in shoots ↔ Ni content in roots	[108]
Pb (2.5 mM Pb(NO_3_)_2_)	0.5 SA	12 h	seeds	*Zea mays*		↑GSH	↑	↓ Pb content ↓ amino acids: Asp, Glu, Asn, Ser, Hist, Gly, Threo, Ala, Ile ↑NR activity ↑protein content	[98]
Cd (500–1000 µM CdCl_2_)	0.5 SA	12 h	seeds	*Triticum aestivum*		↑ SOD, POD, CAT	↑ biomass	↓ Cd content in the leaves and roots ↑proline altered leaf anatomy	[111]
Cd (50–100 µM CdCl_2_)	0.25–1 SA	8 h	seeds	*Linum usitatissimum*	↓ membrane stability			changed profile of membrane lipids, preserved composition of plastidial lipids	[113]
Cd (50–100 µM CdCl_2_)	0.25–1.0 SA	8 h	seeds	*Linum usitatissimum*	↓MDA		↑ biomass, chlorophylls	↓ Cd content ↓ carotenoids ↑total lipid content, altered fatty acid composition altered nutrient distribution between roots and shoots	[114]
Hg (50 µM HgCl_2_)	0.05 SA		2 w-old transplants	*Melissa offcinalis*	↓MDA	↑DPPH, FRAP	↑	↑proline ↑phenolic compounds ↑expression of chlorophyll synthase and PAL	[110]
As (50–100 µM Na_3_AsO_4_)	0.5–1.0 SA	12 h	seeds	*Triticum aestivum*	↓ MDA, H_2_O_2_	↑ SOD, POD ↔ CAT, APX	↑ photosynthetic rate, chlorophylls	↓ As content, also in grains ↑soluble sugars ↑soluble proteins ↑proline	[109]

↓ decrease/reduction; ↑ increase/enhancement,↔ no effect. Abbreviations: APX—ascorbate peroxidase, AsA—ascorbic acid, CAS—cyanoalanine synthase, CAT—catalase, DHA—dehydroascorbic acid, DHAR—dehydroascorbate reductase, DPPH—2,2 diphenyl 1-picrylhydrazyl radical, FRAP—ferric reducing antioxidant power, GDH—glutamate dehydrogenase, GPOX—glutathione peroxidase, GPX guaiacol peroxidase, GR—glutathione reductase, GSH–glutathione (reduced), GSNOR—S-nitrosoglutathione reductase, GSSG—glutathione (oxidized), GST—glutathione S-transferase, LOX—lipooxygenase, MAO—monoamine oxidase, MDA—malondialdehyde, MDHAR—monodehydroascorbate reductase, NPT—nonprotein thiols, NR—nitrate reductase, PAL—phenylalanine amonia-lyase, OAS-TL—O-acetylserine (thiol) lyase, OAT—ornithine aminotransferase, P5CS—delta-1-pyrroline-5-carboxylate synthase, PCs—phytochelatins, POD—peroxidase, ProDH—proline dehydrogenase, PT—protein thiols, ROS—reactive oxygen species, SOD—superoxide dismutase, TBARS—thiobarbituric acid reactive substances, Ve—vitamin E, V-ATPase—vacuolar-type ATPase, XOD—xanthine oxidase.

## Data Availability

Not applicable.

## Supplementary Materials

The following are available online at https://www.mdpi.com/2223-7747/10/4/623/s1, Table S1: Application of metals as priming agents towards secondary non-metallic stress tolerance.
