# Supplementary material for: Priming Strategies for Benefiting Plant Performance under Toxic Trace Metal Exposure
Source: Plants (Basel). 2021 Mar 25;10(4):623. doi: 10.3390/plants10040623 (PMC8064369; doi:10.3390/plants10040623)
Supplement: Supplementary file 1 [file plants-10-00623-s001.pdf]

Table 1. Application of metals as priming agents towards secondary non-metallic stress tolerance.

Priming metal	Plant material	Secondary stress	Effects	Reference
Ag (10–30 mM Ag nano-particles)	*Pennisetum glaucum* seeds	salt stress (120-150 mM NaCl)	↑ biomass<br>↑ proline content<br>↑improved antioxidant defense,<br>↑accumulation of phenolic compounds	[36]
Ca (0.3–0.5 mM $Ca(OH)_2$,Ca(NO3)$_2$, and $CaCl_2$)	*Oryza sativa* seeds	oxidative stress (fluoride induced)	↑ seed germination<br>↑seedling growth<br>↑antioxidant response,<br>↑activity and expression of antioxidant enzymes and proline and glycine betaine synthesis pathway enzymes<br>↓reduced ROS and fluoride accumulation	[38]
Cu (80 µM $CuSO_4$)	*Zea mays* seedlings	biotic stress (insect herbivore attack)	↑ JA synthesis<br>↑volatile organic compounds (VOCs) emission	[39]
Cu (69.4µM nano-Cu°nanoparticles)	*Zea mays* seedlings	drought	↑shoot morphology<br>↑plant biomass and improved grain yield<br>↑ content of chlorophyll, carotenoids and anthocyanins<br>↓ROS production	[32]
K (50 mM KCl)	*Brassica oleracea* seeds	salt stress	↑seed survival rate,<br>↑seedling morphology,<br>↔ nutritional status and phenolic compounds profile	[30]
K (150–300 kg·ha^{-1} K_2O)	*Gossypium hirsutum* plants	drought	↑ fiber strength<br>↑ concentrations of carbohydrates (sucrose, cellulose)<br>↑glucanase activity	[31]
V ($VOSO_4$, 10^{-6} M oxidovanadium(IV) complexes)	*Arabidopsis thaliana* seedlings	oxidative stress (100 mM H_2O_2 treatment)	↑ leaf morphology,<br>plastids and cell walls protected from H_2O_2 accumulation	[37]
Zn (4 mM $ZnSO_4$)	*Zea mays* seeds	salt stress (100 mM NaCl)	↑ biomass<br>↑ nutrient and micronutrient status	[34]
Zn (4 mM $ZnSO_4$), K (23 mM K_2SO_4)	*Zea mays* seeds	salt stress, saline-sodic soil	↑biomass<br>↑elevated shoot Ca and Zn concentrations<br>↑ photosynthesis	[35]
Zn (10 mM NA[1])	*Zea mays* seeds	drought	↑ germination capacity,<br>↑activity of antioxidant enzymes<br>↑ higher Zn translocation to shoots	[33]

↓ decrease/reduction; ↑ increase/enhancement, ↔ no effect. [1] data unavailable
